# Our experience with blast and gunshot induced traumatic vascular injuries at Somalia’s major vascular referral center

**DOI:** 10.1038/s41598-024-63686-5

**Published:** 2024-06-06

**Authors:** Abdinafic Mohamud Hussein, Abdijalil Abdullahi Ali, Said Abdirahman Ahmed, Mohamed Farah Yusuf Mohamud, Mohammed A. M. Ahmed, Mehmet Kizilay

**Affiliations:** 1 Department of Cardiovascular Surgery, Mogadishu Somalia Turkey Recep Tayyip Erdoğan Training and Research Hospital, Mogadishu, Somalia; 2 Department of General Surgery, Benadir University, Mogadishu, Somalia; 3 Department of Cardiology, Mogadishu Somalia Turkey Recep Tayyip Erdoğan Training and Research Hospital, Mogadishu, Somalia; 4 Department of Emergency Medicine, Mogadishu Somalia Turkey Recep Tayyip Erdoğan Training and Research Hospital, Mogadishu, Somalia; 5 Department of Pediatrics, Faculty of Medicine and Surgery, Mogadishu University, Mogadishu, Somalia

**Keywords:** Vascular injuries, Trauma, Gunshot injuries, Blast injuries, Amputation, Cardiology, Diseases

## Abstract

Blast and gunshot-induced penetrating traumatic vascular injuries represent a significant portion of patients with vascular trauma in countries where there are higher rates of war-related violence. These injuries are especially challenging in resource-limited countries due to early diagnosis and transfer delays. This report aimed to present our experience regarding the surgical management and outcome of such injuries at a major referral vascular surgery centre in the country. A retrospective descriptive review of 326 patients with blast and gunshot-induced penetrating traumatic vascular injuries managed during a five-year period between April 2018 and April 2023. The demographics, mechanism of injury, type of vascular injury, Anatomical location, time to the operation, length of hospital stay, amount of blood products given, concomitant neuroskeletal injuries, development of Vascular injury associated acute kidney injury, surgical procedures performed and patient outcome were reviewed. In this study, 326 patients with 445 vascular injuries fulfilled the inclusion criteria. Most of the patients were male 92.3%, and the mean age was 28.3 ± 9.9 years. The gunshot mechanism of vascular injury was implicated in 76.1% of the injuries, and explosive-induced injury was 78 (23.9%). 193 (59.2%) of the patients had isolated arterial injuries, 117 (35.9%) patients had combined arterial and venous injuries while 18 (4.9%) patients had isolated venous injuries. The most commonly injured arteries were the femoral artery, followed by Brachial and popliteal artery injuries (26.1%, 23.5% and 19.4%, respectively). The median time to revascularization was 8.8 ± 8.7 h. 46.8% of the patients had Concomitant fractures, while 26.5% had Concomitant nerve injuries. Only three patients had temporary non-heparin-bound shunts during their arrival. The most common surgical intervention in arterial injuries was reversed saphenous vein graft 46.1%. The mortality was 5.8% and 7.7% of the patients needed secondary amputation. The majority of wartime arterial injuries are a result of Blast and gunshot vascular injuries. Frequent need for autologous vein grafts should be considered to manage such injuries. Results are encouraging despite delays in intervention; therefore, all viable limbs should be revascularized, keeping in mind the long-term functionality of the limb.

## Introduction

Traumatic vascular injuries are considered infrequent but can cause significant morbidity and mortality in both civilian and military populations^1^. Due to higher rates of terror and civil war-related violence in the population, blast and gunshot-induced traumatic injuries are common in Somalia^2^. The conflict in Somalia has been dating back since the collapse of Siaad Barre’s Government with varying degrees of intensity and dynamics throughout the years, with the root causes of conflict considered to be mainly terrorism and inter-clan conflicts^3^.

Blast injuries result in devastating combinations of injuries that can affect multiple body systems. They cause injuries through high-pressure blast waves, bomb fragments, violent displacement, collapse of infrastructure, and heat. Blast explosions in Somalia are mainly landmines, grenades and improvised explosive devices^2,4^. The nature of gunshot injury is determined by the anatomic location involved, the dynamics of the projectile and the local reaction of the penetrated tissue^5^.

The mechanism and pattern of traumatic vascular injuries vary within the community in peace and war. In civilian practice, road accidents are considered the most common cause, but in countries with armed conflicts, penetrating traumas due to stab wounds, gunshots, and blast explosions are the most common cause of vascular injuries^6^. Surgical management of traumatic vascular injuries has evolved over time from where Ligation was the principal surgical strategy during World War I to an era where reversed vein grafts and prosthetic vascular grafts became widely used^7^.

To date, bullet and blast-induced traumatic vascular injuries have been studied in different parts of the world^6^. However, the anatomical distributions, nature and management of vascular injuries affecting casualties in Somalia, where explosions and gunshot-related violence are usually intense, have not been studied in detail.

Our hospital is located in Mogadishu, the capital city of Somalia. It was opened based on a protocol signed by the health ministries of the two countries Somalia and Türkiye to develop the cooperation between the countries in the field of health and medicine. The hospital is a major referral vascular surgery Centre for the country, where patients from Mogadishu and other parts of the country with traumatic vascular injuries are referred. In this study, we aimed to share our experience on anatomic distribution, associated injuries, surgical treatment techniques, and the outcomes of Bullet and blast-induced traumatic vascular injuries that were treated in our hospital over a period of five years.

## Method

This study was designed as a retrospective descriptive study of 5 years between April 2018 and April 2023 conducted in the Department of Vascular Surgery of Mogadishu, Somalia, Turkey, Recep Tayyip Erdogan Research and Training Hospital. The data of this article was retrieved from the FONET electronic system of the hospital and is available from the corresponding author on reasonable request. It was reviewed retrospectively by a single investigator, and medical data of all patients with traumatic vascular injuries was collected by single investigator. Patients with blast and gunshot-induced traumatic vascular injuries who had undergone revascularization were included in the study, while patients with other causes of traumatic vascular injuries or with digital artery injuries were excluded from the study. Patients who had non-salvageable traumatic limb loss or those who needed lifesaving primary amputations were excluded from the study. Ethical approval was obtained from the hospital's ethics committee with reference number (MSTH/4776) and was performed in accordance with relevant guidelines and regulations. Informed consent was obtained from all participants and/or their legal guardians.

Data collected included age, gender, mechanism of injury, type of vascular injury, Anatomical location, timing (from the point of wounding to the operation table), length of hospital stay, amount of blood products given, concomitant neuroskeletal injuries, development of Vascular injury associated acute kidney injury, surgical procedures performed and patient outcome (Recovery, Secondary amputation and death).

Patients were initially resuscitated according to Advanced Trauma Life Support guidelines. diagnosis of vascular injury was achieved through a physical examination with the assistance of hand Doppler, colour flow Doppler ultrasound and computed tomography angiography on patients with persistent pulse discrepancies and hemodynamic stability or surgical exploration for patients with hard signs of arterial injury^8^. Associated bone fractures were assessed with relevant X-rays where indicated. The decision to proceed with vascular repair rather than primary amputation when patients came after 6 h of injury, and patients could not move their toes or ankle was based on the viability of the distal muscle by open fasciotomy and observation of the contractile response of muscle to direct stimulation.

Standard surgical techniques for revascularization were used. After the incision, Proximal and distal control was obtained. A bolus of 100 IU/kg of heparin was administered. Inflow and backflow were assessed, and a thrombectomy was performed. This was followed by definitive repair with end-to-end anastomosis, Primary repair, interposition of Great saphenous vein graft, Cephalic or basilic vein graft interposition in a few cases with brachial artery injury, or Polytetrafluoroethylene grafts. Orthopaedic fixation was performed after vascular repair to reduce the duration of ischaemia if there were accompanying bone injuries. Nerve injuries identified were primarily repaired.

For the purpose of this study, Explosive injury was defined as acute injury arising from a blast mechanism with penetrating explosive fragments (i.e. secondary blast injury). Gunshot-related injury was defined as when there was acute physical injury caused by gunshots. Primary amputation was defined as amputation performed without any attempt revascularization due to traumatic limb loss or those who needed lifesaving immediate amputations. Secondary amputation was defined as amputation performed after an attempt of limb salvage. The presence of Vascular injury associated kidney injury was defined as an abrupt (within 48 h) reduction in kidney function, diagnosed with an absolute increase in Serum creatinine of more than or equal to 26.4 μmol/l^9^.

The statistical analysis of this study was performed with the use of Statistical Package for Social Sciences Version 23.0 software (SPSS Inc., Chicago, IL, USA). Variables were compared by using Chi-square analysis or the Fisher exact test. *P* values ≤ 0.05 were considered statistically significant.

### Ethical approval

Ethical approval and consent for publication was obtained from the hospital's ethics committee with reference number (MSTH/4776).

## Results

In a period of 5 years, a total of 458 patients with traumatic vascular injuries were managed in our hospital. Among these patients, 326 patients had blast, and gunshots induced traumatic vascular injuries and were candidates for the inclusion criteria. 301 (92.3%) of our study population were male while 25 (7.7%) were female. The age of the patients ranged from 7 to 66 years old, and the mean age of 28.3 ± 9.9 years. The gunshot mechanism of vascular injury was implicated in most of the cases, 248 (76.1%), and explosive-induced injury was 78 (23.9%). The mean time from the incident to the operation table was 8.8 ± 8.7 h, and the length of hospital stay was 10.3 ± 9.0 days. Demographic data are summarized in Table 1.

**Table 1:** Patient demographics data.

Patient demographics	Number	%
Age (years)	28.3 ± 9.9	
Gender		
Male	301	92.3%
Female	25	7.7%
Mechanism		
Gunshot injury	248	76.1%
Blast injuries	78	23.9%
Blood products	8.4 ± 8.3	
Hospital stay (days)	10.3 ± 9.0	
Time to the operation room (h)	8.8 ± 8 .7	

There were 445 vascular injuries in these 326 patients. 193 (59.2%) patients had isolated arterial injuries, 117 (35.9%) patients had combined arterial and venous injuries while 18 (4.9%) patients had isolated venous injuries. The Anatomical distribution of arterial injuries is summarized in Table 2, revealing that the femoral artery is the most common artery injured, followed by Brachial and popliteal artery injuries (26.1%, 23.5% and 19.4%, respectively). Venous injuries are summarized in Table 3.

**Table 2:** The anatomical distribution, Associated acute Kidney injury, Neuroskeletal Injury and outcome of arterial injuries.

Arterial injury n = 310	Associated acute kidney injury n = 23		Neuroskeletal Injury		Outcome n = 310		
			Fracture n = 145	Nerve injury n = 82	Recovery	Secondary amputation	Death
Carotid artery	12 (3.9%)	–	–	–	10	0	2
Subclavian Artery	6 (1.9%)	–	1 (0.7%)	4 (4.9%)	6	0	0
Axillary artery	15 (4.8%)	–	1 (0.7%)	11 (13.4%)	15	0	0
Brachial artery	73 (23.5%)	4 (17.4%)	32 (22.1%)	17 (20.7%)	71	0	2
Radial/Ulnar artery	20 (6.5%)	–	12 (8.3%)	10 (12.2%)	20	0	0
Iliac Artery	3 (1.0%)	1 (4.4%)	–	–	3	0	0
Femoral artery	81 (26.1%)	7 (30.4%)	25 (17.2%)	17 (20.7%)	65	8	8
Popleteal artery	60 (19.4%)	11 (47.8%)	42 (28.9%)	15 (18.3%)	40	14	6
Infrapopliteal arteries	40 (12.9%)	–	32 (22.1%)	8 (9.8%)	38	2	0
Total	310 (100%)	23 (100%)	145 (100%)	82 (100%)	268 (86.5%)	24 (7.7%)	18 (5.8%)

**Table 3:** Anatomical distribution of venous injuries.

	N	%
Jegular vein	3	2.2
Subclavian vein	1	0.7
Axillary vein	8	5.9
Basilic/Cephalic/Brachia vein	30	22.2
IVC	5	3.7
Iliac vein	6	4.5
Femoral vein	47	34.8
Popliteal vein	35	26.0
Total	135	100

In patients with arterial injuries, 145 (46.8%) patients had Concomitant fractures and needed orthopaedic intervention, while 82 (26.5%) patients had Concomitant peripheral nerve injuries. The distribution of fracture and nerve injuries according to the anatomical distribution of arterial injuries can be seen in Table 2.

As shown in Table 4, the primary surgical interventions undertaken in arterial vascular injuries were Reversed Saphenous vein graft (n = 310 [46.1%]) followed by End-to-end anastomosis (n = 310 [39.7%]). Venous vascular injuries were managed mainly with Primary repair (n = 135 [37.0%]) and Reversed Saphenous vein graft (n = 135 [18.5%]). Five patients with common femoral and iliac venous injuries were managed with Spiral saphenous vein grafts. Only three patients came to the hospital with temporary non-heparin-bound shunts, although none of them were patent during their arrival. Surgical interventions of the vascular injuries were conducted by Cardiovascular Surgery specialists and the residents' team.

**Table 4:** Surgical treatment modalities for vascular injuries.

	Arterial injuries n = 310	Venous injuries n = 135
Primary repair	13 (4.2%)	50 (37.0%)
End-to-end anastomosis	123 (39.7%)	19 (14.1%)
Reversed Saphenous vein graft	143 (46.1%)	25 (18.5%)
Spiral saphenous vein graft	–	5 (3.7%)
Cephalic vein graft	3 (1.0%)	–
Patch repair	–	11 (8.2%)
Synthetic graft	12 (3.9%)	5 (3.7%)
Ligation	16 (5.1%)	20 (14.8%)
Total	310 (100.0%)	135 (100.0%)

Postoperative follow-up of the patients revealed that 268 (86.5%) of the patients with arterial injury recovered, 24 (7.7%) of the patients underwent secondary amputation, and 14 (5.8%) of the patients Died. Popliteal artery injury, followed by femoral artery injury, was associated with most of the amputations and deaths. 14 (23.3%) of the patients with popliteal artery injury underwent secondary amputation, while 6 (10.0%) died. On the other hand, Secondary amputation or mortality of the patients with femoral artery injury was 8 (10.0%). In terms of upper limb vascular injuries, only two patients with brachial artery injuries died, and one of these patients had a massive epidural hematoma.

The overall prevalence of acute kidney injury in this study was 24(7.4%), and most of the AKI’s were associated with popliteal and femoral artery injuries, 47.8% and 30.4%, respectively. There was a strong association between the number of blood products transfused and the development of acute kidney injury (*p* < 0.001).

## Discussion

In many parts of the world, trauma has emerged as a major public health issue, with vascular trauma playing a significant role. Penetrating traumas, like stab, gunshot wounds, and blast injuries, as well as traffic accidents, account for a large percentage of these wounds^10^. According to our analysis, the majority of the patients with traumatic vascular injury are victims of fragment wounds from explosive devices or gunshot wounds due to higher rates of terror and civil war-related violence in Somalia, with most of them being males in their twenties.

The femoral artery, followed by Brachial and popliteal artery injuries, were the most common injured arteries in our study. This is similar to the pattern of war-related vascular injuries reported by Jawas et al.^6^.

Vascular structures often lie in close proximity to nerves and bones; associated skeletal and nerve injury occurred in 44.5 and 25% of the patients, respectively. Most of the fractures were related with popliteal artery injury followed by infra-popliteal and brachial artery injuries. The rate of skeletal injury in our study is high compared to other reports, and the reason for that could be the high-kinetic energy mechanism of such injuries^11,12^.

The extent of vascular injury determines the best course of vascular restoration. Most of the blast and gunshot-exposed arterial injuries had a segmental loss of > 2 cm and needed Reversed Saphenous vein graft in 46.1% of the cases. The frequent use of autologous vein grafts in wartime arterial injuries has been supported by many studies in the literature ^7,13^.

For most of the first half of the twentieth century, regular venous ligation had been the standard procedure of venous injury management. However, the importance of venous restoration was later highlighted to prevent a low-flow state, with particular attention to the popliteal region^14^. Venous ligation was only 14.8% in our experience, and most of the venous injuries were managed with primary venous repair. It is possible that the aggressive management of venous injuries contributed to a good outcome despite our patients' delayed presentation. Concomitant Femoral, followed by popliteal arterio-venous injuries, were the most common types of vascular injury, with the highest mortality and secondary amputation.

The most lethal arterial injury in our series is the popliteal artery, which was associated with 58.3% of the secondary amputations and 33.3% of the deaths among the patients. Injury to this artery was also responsible for 47.8% of vascular injuries associated with acute kidney injury. In multiple studies, injury to this vessel was recognized as the most difficult and dangerous injury and was associated with the highest rate of limb loss^15–17^. The unique anatomy of this vessel, the difficulty of surgical exposure, and commonly associated injuries like venous injury, fracture, nerve injury, and other associated soft tissue injuries contribute to its surgical challenge^17^.

Our study shows a secondary amputation rate of 24 (7.7%) and death of 14 (5.8%). Due to absence of well-organized casualty care, limited facilities and expertise to manage vascular injuries in different parts of Somalia coupled with delays in early diagnosis and transfer, led to an increase of time between the incident areas to the operation table.

Despite delayed revascularization with a time to operation of 8.8 h, the overall limb salvage and survival rate after vascular repair is remarkable in this series and compares with other recent reports. Iraj and Velinovic reported a secondary amputation rate of 6.9% and 12.3% respectively, and Fahad et al. reported a mortality of 5.48% in their 10 years' experience of vascular injuries in a single centre in Karachi, Pakistan^12,18,19^.

Patients with vascular trauma are at risk of acute kidney injury caused by renal hypoperfusion (secondary to haemorrhagic shock), rhabdomyolysis, or the nephrotoxic effects of therapies^20^. The incidence of post-traumatic AKI varies widely in different studies; in our study the rate of AKI after Vascular injury was 7.4%. Anatole reported the overall incidence of acute kidney injury among trauma patients in the multicenter trauma registry as 13%, but the incidence rose up to 42.5% in patients presenting with hemorrhagic shock^20^. We have seen that there was a strong association between the numbers of blood products transfused with the development of acute kidney injury.

We did not encounter a significant number of acute kidney injury, Secondary amputation and mortality in this series despite our approach to revascularize all viable limbs with long periods of ischemia. This suggests that delayed revascularization should not be discouraged based only on ischemia time. The severity of tissue ischemia depends not only on its duration but also on the efficiency of collateral circulation, the level of arterial injury and the extent of soft tissue damage^21^. De Silva et al. outlined that they did not encounter clinically significant systemic effects from reperfusion in their series of seventy patients despite accepting patients with long periods of ischemia and with non-contractile muscles in up to two compartments^22^. It is also important to emphasize that limb salvage is not enough on its own, but the long-term functionality of the limb must be taken into account, which frequently depends on the severity and rate of recovery from related neuromuscular and skeletal impairments. Based on this experience, we also highlight the need for organized casualty care and vascular surgeons or trauma surgeons who can perform vascular surgery in low-income countries where war-related vascular injuries are common.

Although promising, this study contained several limitations. In addition to the retrospective design, we were unable to follow up on the long-term functionality of the limbs of patients who presented late, but their limbs survived and were discharged due to their low socioeconomic status and lack of health insurance. Despite this, our study presents a comprehensive work describing the nature and challenges of blast and gunshot-induced traumatic vascular injuries in a population where such injuries are common.

## Data Availability

The data of this article was retrieved from the FONET electronic system of the hospital and is available from the corresponding author on reasonable request.

## References

[1] He JC, Clancy K, Schechtman D, Conrad-Schnetz KJ, Claridge JA (2016). Traumatic vascular injuries: Who are repairing them and what are the outcomes?. Am. J. Surg..

[2] Tahtabasi M, Er S, Karasu R (2021). Bomb blast: Imaging findings, treatment and clinical course of extremity traumas. BMC Emerg. Med..

[3] Bade ZA (2021). Understanding Somali conflict: Causes, consequences and strategies for peace-building. Dev. Country Stud..

[4] Plurad DS (2011). Blast injury. Mil. Med..

[5] Stefanopoulos PK, Hadjigeorgiou GF, Filippakis K, Gyftokostas D (2014). Gunshot wounds: A review of ballistics related to penetrating trauma. J. Acute Dis..

[6] Jawas A, Abbas AK, Nazzal M, Albader M, Abu-Zidan FM (2013). Management of war-related vascular injuries: Experience from the second gulf war. World J. Emerg. Surg..

[7] Sharrock AE, Tai N, Perkins Z, White JM, Remick KN, Rickard RF (2019). Management and outcome of 597 wartime penetrating lower extremity arterial injuries from an international military cohort. J. Vasc. Surg..

[8] American College of Surgeons. ATLS Course Manual. *Advanced Trauma Life Support Course Manual*. 48–61 (2018).

[9] Id ZBP, Captur G, Bird R, Gleeson L, Singer B, Brien BO (2019). Trauma induced acute kidney injury. PLoS ONE.

[10] Murad M, Eweda A, Abdel-Moamen H, Hussien M, Elsaghir M (2013). Vascular trauma and its management: One and a half years after the 25th January revolution. Arab. Soc. Med. Res..

[11] Fox CJ, Gillespie DL, O’Donnell SD, Rasmussen TE, Goff JM, Johnson CA (2005). Contemporary management of wartime vascular trauma. J. Vasc. Surg..

[12] Khan FH, Yousuf KM, Bagwani AR (2015). Vascular injuries of the extremities are a major challenge in a third world country. J. Trauma Manag. Outcomes.

[13] Dhillan R, Bhalla A, Jha SK, Singh H, Arora A (2019). Vascular injuries due to penetrating missile trauma in anti-Terrorism Ops. J. Trauma Inj..

[14] Ratnayake AS, Samarasinghe B, Bala M (2017). Challenges encountered and lessons learnt from venous injuries at Sri Lankan combat theatres. J. R. Army Med. Corps..

[15] Sciarretta JD, Perez-Alonso AJ, Ebler DJ, Mazzini FN, Petrone P, Asensio-Gonzalez JA (2012). Popliteal vessel injuries: Complex anatomy, difficult problems and surgical challenges. Eur. J. Trauma Emerg. Surg..

[16] Shi L (2012). The delayed management of main arterial injuries in extremity trauma: Surgical challenges and outcomes. Pak. J. Med. Sci..

[17] Asensio JA, Dabestani PJ, Miljkovic SS, Kotaru TR, Kessler JJ, Kalamchi LD (2020). Popliteal artery injuries. Less ischemic time may lead to improved outcomes. Injury.

[18] Baghi I, Herfatkar MR, Shokrgozar L, Poor-Rasuli Z, Aghajani F (2015). Assessment of vascular injuries and reconstruction. Trauma Mon..

[19] Velinovic MM, Davidovic BL, Lotina IS, Vranes RM, Djukic LP, Arsov JV (2000). Complications of operative treatment of injuries of peripheral arteries. Cardiovasc. Surg..

[20] Harrois A, Soyer B, Gauss T, Hamada S, Raux M, Duranteau J (2018). Prevalence and risk factors for acute kidney injury among trauma patients: A multicenter cohort study. Crit. Care.

[21] Hafez HM, Woolgar J, Robbs JV (2001). Lower extremity arterial injury: Results of 550 cases and review of risk factors associated with limb loss. J. Vasc. Surgery.

[22] De SWDD, Ubayasiri RA, Weerasinghe CW, Wijeyaratne SM (2011). Challenges in the management of extremity vascular injuries: A wartime experience from a tertiary centre in Sri Lanka. World J. Emerg. Surg..

